# Supplementary material for: Joint-level energetics differentiate isoinertial from speed-power resistance training—a Bayesian analysis
Source: PeerJ. 2018 Apr 12;6:e4620. doi: 10.7717/peerj.4620 (PMC5899884; doi:10.7717/peerj.4620)
Supplement: Supplemental Information 2 [file peerj-06-4620-s002.pdf]

Figure S1 CMJ height

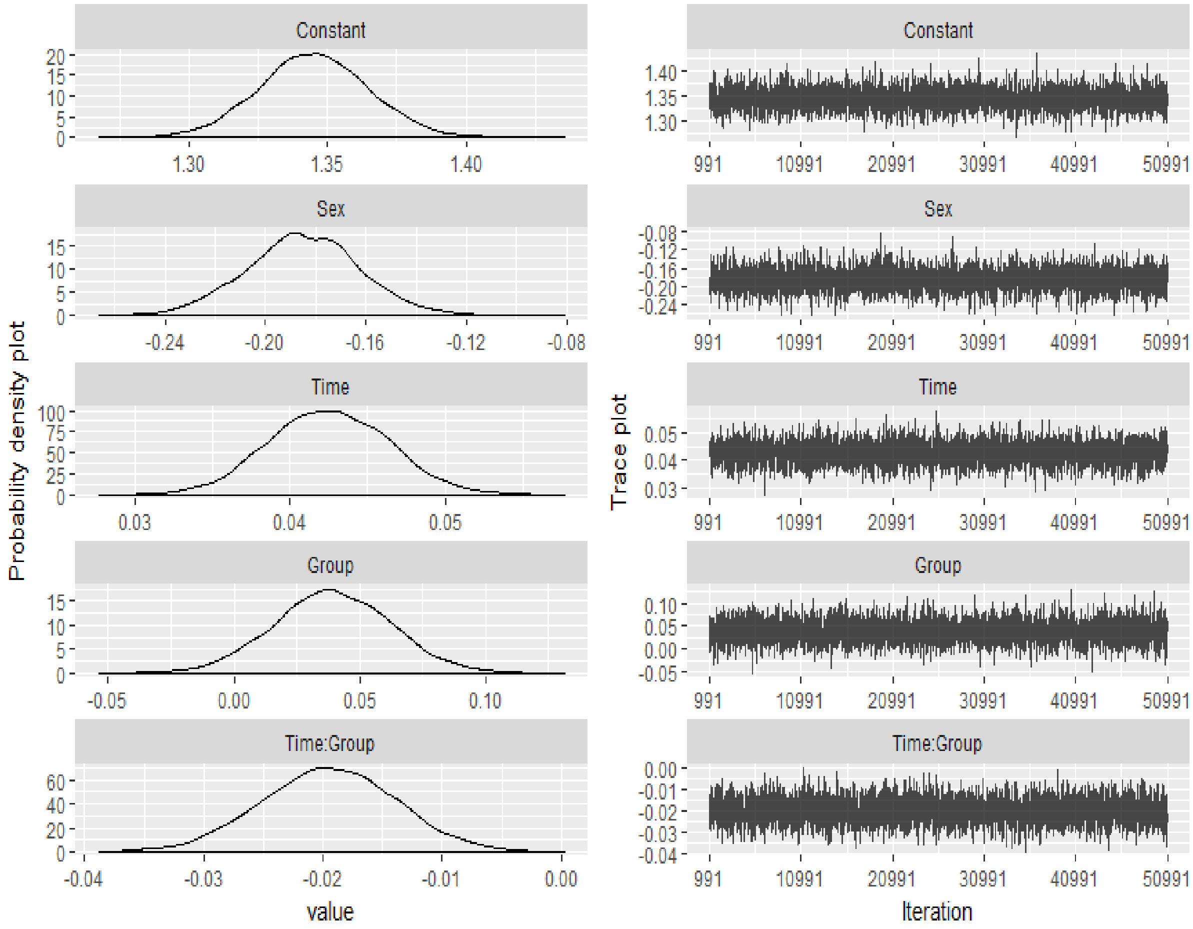

Figure S2 SJ height

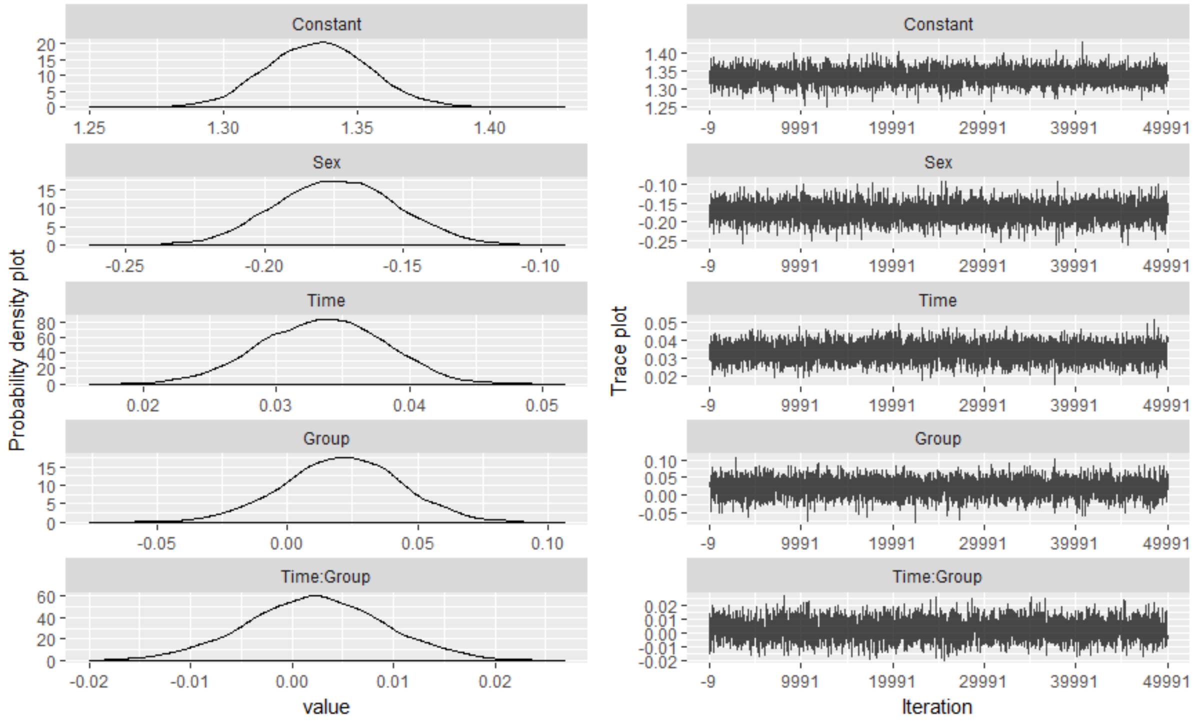

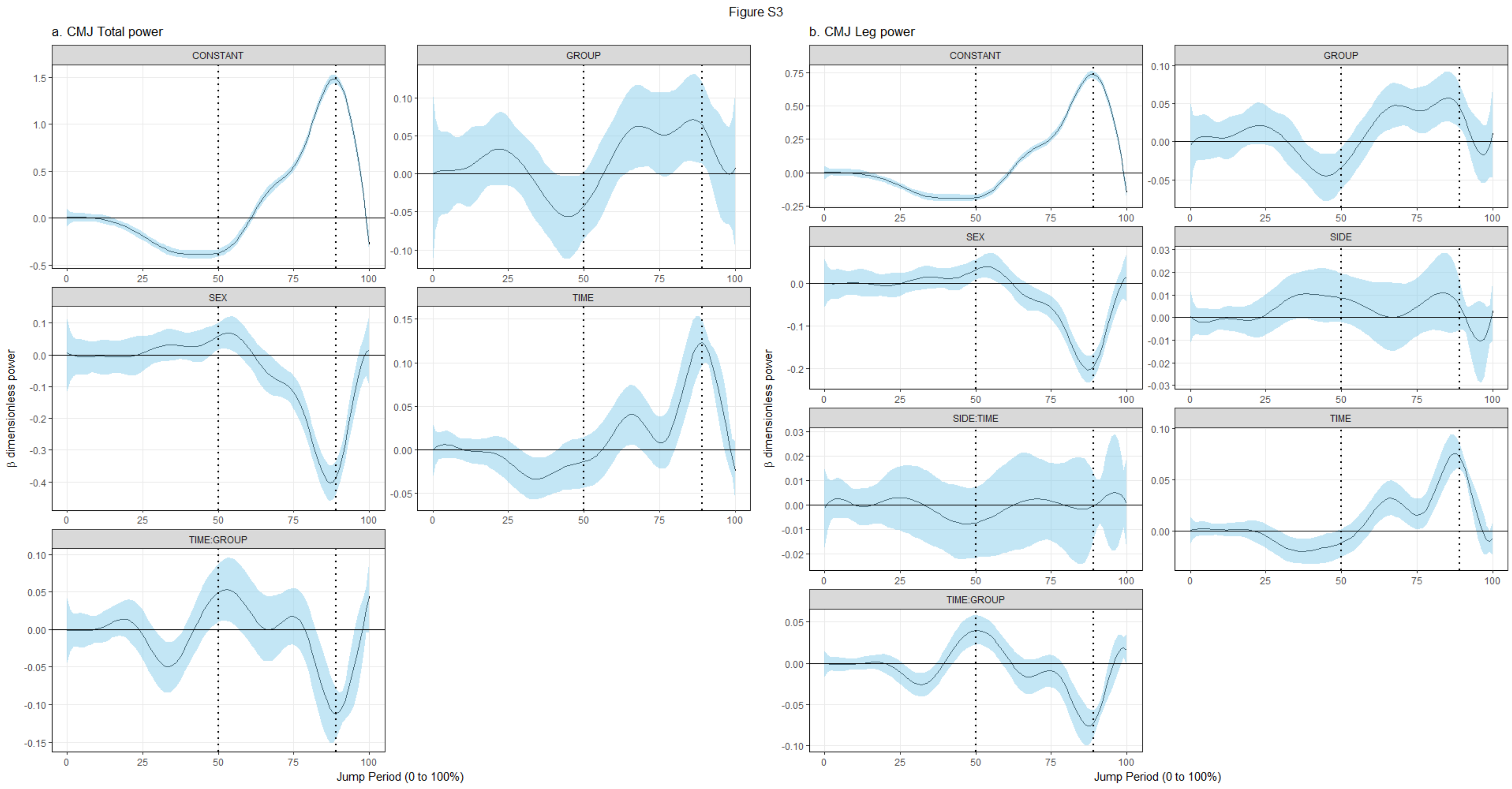

Figure S3. Mean (95% credible intervals as error clouds) of regression coefficients of joint power in countermovement jump

Figure S3 Continued

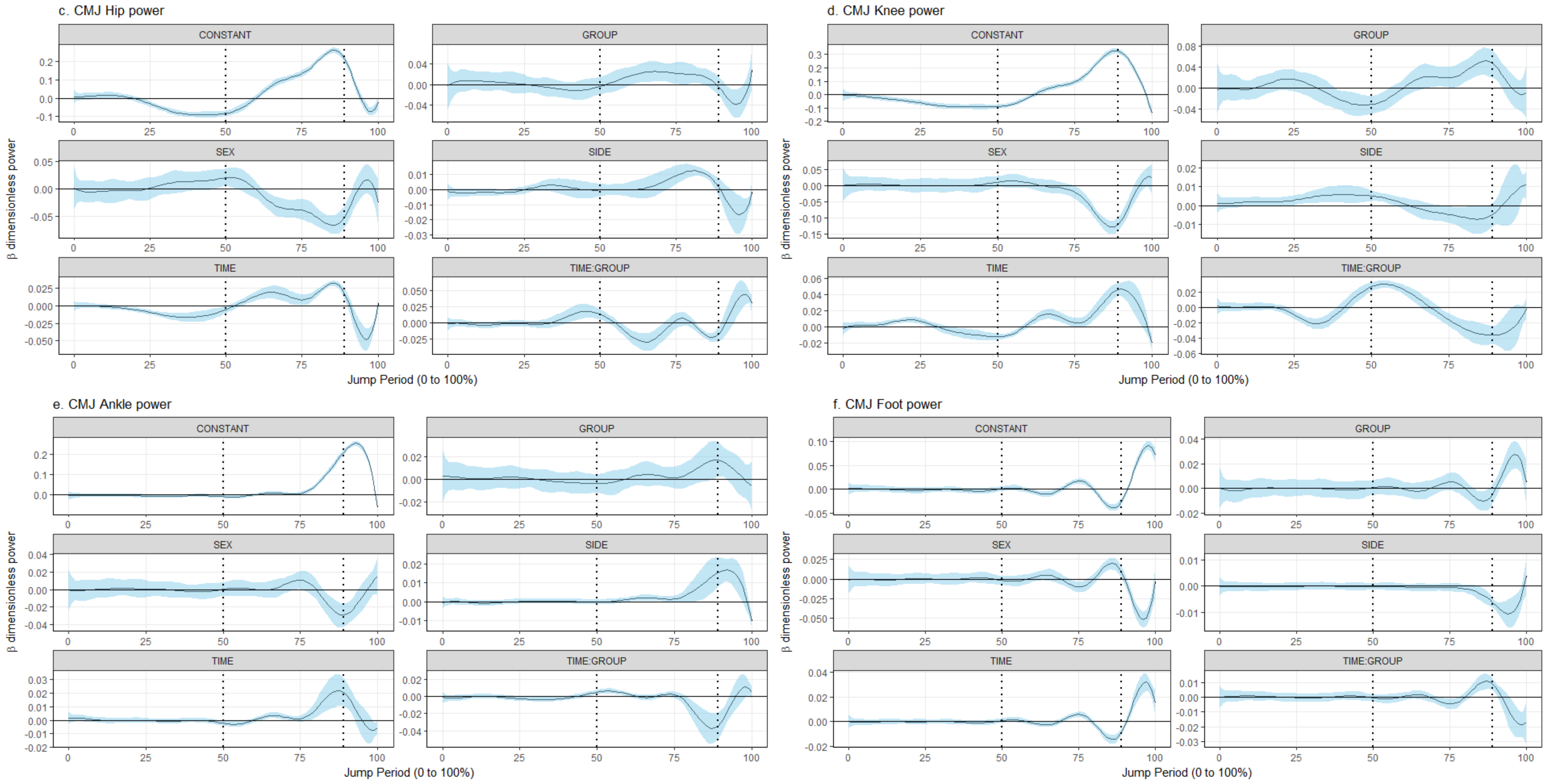

Figure S3. Mean (95% credible intervals as error clouds) of regression coefficients of joint power in countermovement jump

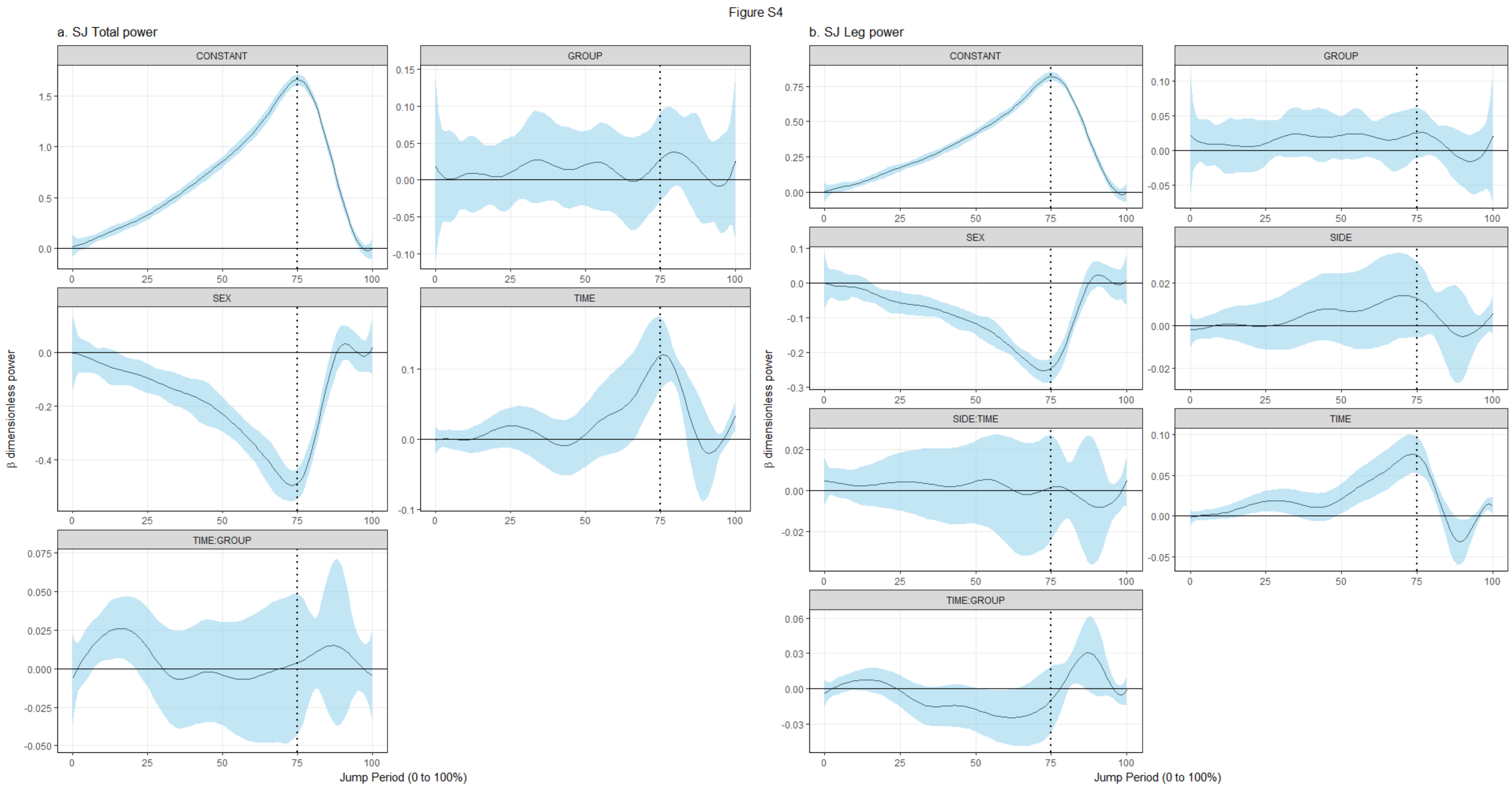

Figure S4. Mean (95% credible intervals as error clouds) of regression coefficients of total and leg power in squat jump

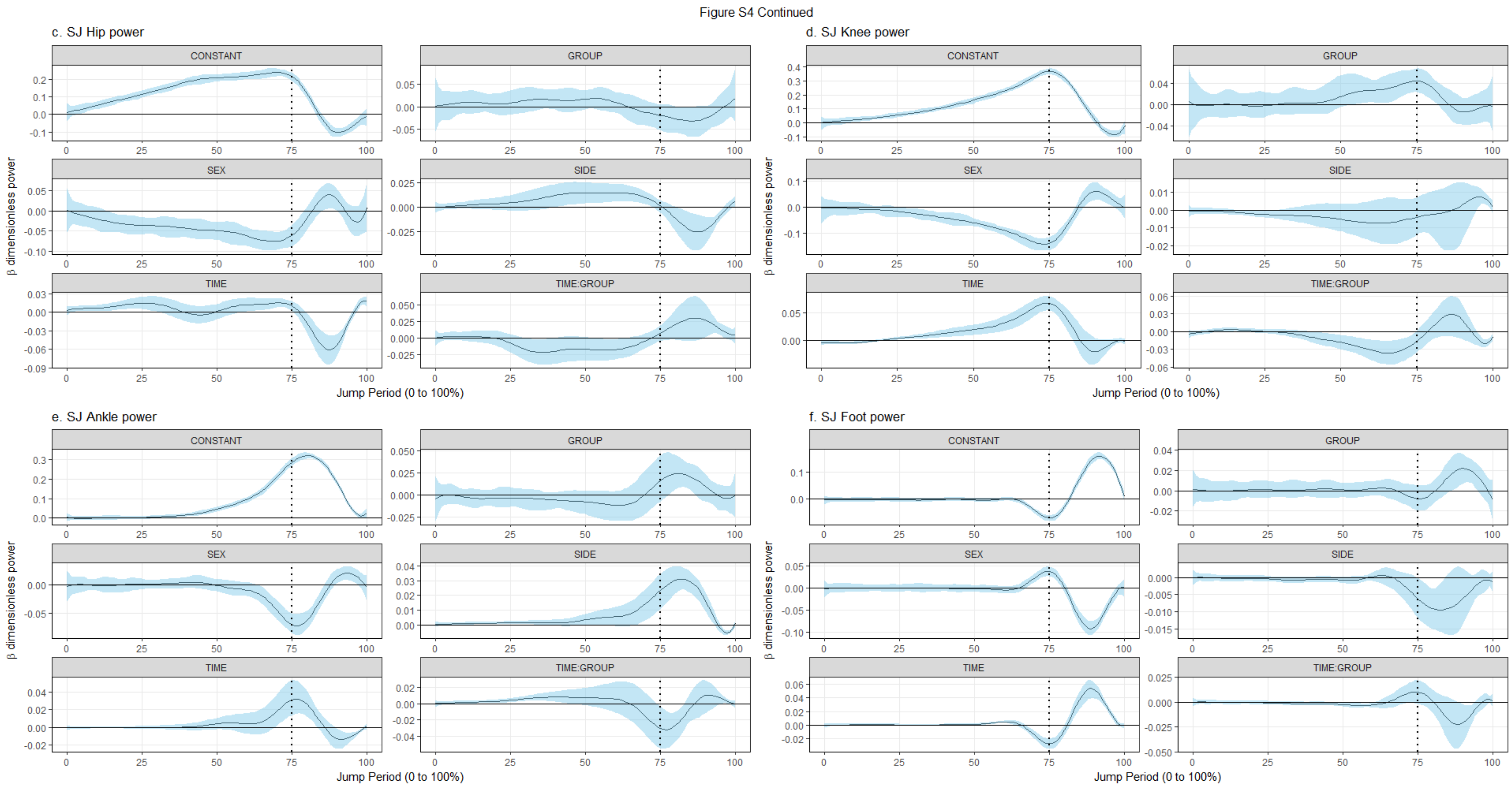

Figure S4. Mean (95% credible intervals as error clouds) of regression coefficients of joint power in squat jump
